# The Effect of Two Interventions to Increase Breast Cancer Screening in Rural Women

**DOI:** 10.3390/cancers14184354

**Published:** 2022-09-07

**Authors:** Victoria L. Champion, Patrick O. Monahan, Timothy E. Stump, Erika B. Biederman, Eric Vachon, Mira L. Katz, Susan M. Rawl, Ryan D. Baltic, Carla D. Kettler, Natalie L. Zaborski, Electra D. Paskett

**Affiliations:** 1 School of Nursing, Indiana University, Indianapolis, IN 46202, USA; 2 Indiana University Melvin and Bren Simon Comprehensive Cancer Center, Indianapolis, IN 46202, USA; 3 Department of Biostatistics and Health Data Science, Indiana University School of Medicine, Indianapolis, IN 46202, USA; 4 Comprehensive Cancer Center, The Ohio State University (OSU), Columbus, OH 43210, USA; 5 Division of Health Behavior and Health Promotion, The Ohio State University, Columbus, OH 43210, USA; 6 Division of Cancer Prevention and Control, Department of Medicine, College of Medicine, The Ohio State University, Columbus, OH 43210, USA

**Keywords:** mammography, intervention, rural, breast cancer screening, cancer screening

## Abstract

**Simple Summary:**

This research tested the effectiveness of a mailed DVD and mailed DVD + PN (patient navigation) to increase mammography screening compared to usual care in rural women. Women in the combined DVD + PN were over 5 times more likely to have received a mammogram 12 months after the intervention.

**Abstract:**

Guideline-based mammography screening is essential to lowering breast cancer mortality, yet women residing in rural areas have lower rates of up to date (UTD) breast cancer screening compared to women in urban areas. We tested the comparative effectiveness of a tailored DVD, and the DVD plus patient navigation (PN) intervention vs. Usual Care (UC) for increasing the percentage of rural women (aged 50 to 74) UTD for breast cancer screening, as part of a larger study. Four hundred and two women who were not UTD for breast cancer screening, eligible, and between the ages of 50 to 74 were recruited from rural counties in Indiana and Ohio. Consented women were randomly assigned to one of three groups after baseline assessment of sociodemographic variables, health status, beliefs related to cancer screening tests, and history of receipt of guideline-based screening. The mean age of participants was 58.2 years with 97% reporting White race. After adjusting for covariates, 54% of women in the combined intervention (DVD + PN) had a mammogram within the 12-month window, over 5 times the rate of becoming UTD compared to UC (OR = 5.11; 95% CI = 2.57, 10.860; *p* < 0.001). Interactions of the intervention with other variables were not significant. Significant predictors of being UTD included: being in contemplation stage (intending to have a mammogram in the next 6 months), being UTD with other cancer screenings, having more disposable income and receiving a reminder for breast screening. Women who lived in areas with greater Area Deprivation Index scores (a measure of poverty) were less likely to become UTD with breast cancer screening. For rural women who were not UTD with mammography screening, the addition of PN to a tailored DVD significantly improved the uptake of mammography. Attention should be paid to certain groups of women most at risk for not receiving UTD breast screening to improve breast cancer outcomes in rural women.

## 1. Introduction

Breast cancer remains the most frequently diagnosed cancer in women, with only lung cancer ranking higher in cancer mortality [1]. Although breast cancer screening by mammography has been promoted for the last three decades, the percentage of women having a mammogram in 2018 compared to 2000 has decreased in women 40 and older from a high of 70.4% in 2000 to 65.6% in 2018 [2]. Additionally, although recent data observed similar mammography rates in rural and urban areas of the United States, mammography rates in rural Ohio and Indiana are lower than national rates [3]. If we are to reach the Healthy People 2030 goals of 77.1% breast cancer screening adherence [4], interventions to increase mammography screening must be implemented with particular emphasis in underserved rural areas.

The Social Determinants of Health for women in rural areas include lower rates of a college degree [5], higher rates of poverty [6,7,8], and lower rates of insurance coverage [9]. Women residing in rural areas also face logistical barriers to accessing health care services, including a sparsity of sites offering mammography screening. Longer traveling distances to mammography services, often experienced by women in rural settings, are associated with lower rates of utilization [10,11]. Rural women also have more fatalistic beliefs about breast cancer which potentially decreases their motivation to engage in screening behaviors [3]. As a result, compared to women in urban areas, fewer women living in rural areas are up to date (UTD) with mammography [5].

Interventions to increase population-based mammography screening have been developed and tested for over two decades and include technology-based interventions supported by tailored messaging [12,13,14,15,16,17,18,19,20]. Additionally, patient navigation (PN) has been shown to increase women’s motivation and self-efficacy to complete mammography screening by reducing and/or removing barriers to screening [21,22]. Furthermore, research has demonstrated that combining education or tailored messaging with PN increases the likelihood of participants to complete mammography screening [23].

Although interventions using tailored messaging and PN have significantly increased mammography screening compared to usual care, few studies have been designed and adapted specifically for delivery in rural regions. Building on past research, we combined tailored messaging within an interactive DVD and telephone based PN to deliver a home-based intervention focused on increasing breast cancer screening to women living in rural Indiana and Ohio. This comparative effectiveness trial randomized women to usual care (UC), a tailored interactive cancer screening program using a DVD format, or the combination of the DVD plus PN (DVD/PN). The parent intervention trial sought to simultaneously increase the completion of three cancer screenings recommended for women- breast, cervical and colorectal (CRC). This report focuses on the outcome of breast cancer screening and is framed by three aims. First, we sought to determine if there was a difference in mammography at 12 months post intervention (T3) by randomized arm while controlling for covariates. Secondly, we tested for an interaction between the interventions and baseline variables. Finally, using data from the larger study, we explored the association between the type of cancer screening needed at baseline and being UTD at T3 with breast cancer screening.

## 2. Materials and Methods

### 2.1. Sample

From 2016–2019 women were recruited from the 98 rural Indiana and Ohio counties with a *Rural-Urban Continuum Code (RUCC*) ranging from 4–9 [24]. Eligibility criteria for this analysis included: (1) ages 50–74 years; (2) not UTD for guideline-based breast cancer screenings; and (3) able to speak English. The definition for being UTD with breast cancer screening included having a mammogram within the last two years [25]. Screening was verified via Medical Record Review (MRR) and was used to both confirm baseline eligibility and determine screening outcomes at T3. Women were ineligible if they had a previous cancer diagnosis, other than skin cancer.

Recruitment of women included the following three methods: (1) a commercial list of age-eligible women residing in rural counties in Indiana and Ohio; (2) personal contact through community events, and (3) an invitation to eligible women on Facebook and Craigslist. Eligible participants were randomized to one of three groups: (1) mailed DVD; (2) mailed DVD + PN, or (3) UC.

### 2.2. Interventions

DVD: An interactive, tailored DVD was developed to provide information for any cancer screening-breast, cervical or CRC-for which participants were not UTD. The DVD was mailed to each participant assigned to both intervention arms (DVD and DVD + PN). Participants who were not UTD with breast cancer screening guidelines were directed to view the tailored mammography screening program included in the DVD. The DVD was based on a previous efficacious technology-based screening intervention developed by study investigators [26]. Participants used a DVD remote control to answer questions presented on menus within the DVD, and a tailored algorithm provided appropriate messages based on individual inputted responses. Content for interactive messages within the DVD were supported by a theoretical framework and were revised from an extensive message library used in prior research [15,16,17,18,19,20]. Tailored messages were delivered based on each user’s cancer screening history; knowledge of and risk factors for breast cancer; perceived benefits and barriers to breast cancer screening; and self-efficacy to obtain breast cancer screening. Information about scheduling and completing breast cancer screening was also included in the DVD.

Patient Navigation: A patient navigator called women randomized to the DVD + PN intervention group one week after the DVD was mailed. PNs were licensed social workers trained by study investigators and staff. Once they were able to contact the participant, navigators assessed breast cancer screening knowledge and barriers and provided information about the benefits of breast cancer screening, as well as information about available transportation to a clinic, if needed. The PN intervention fidelity was assessed from 10 randomly selected recorded calls per month.

### 2.3. Measures

Self-report measures, obtained at baseline and T3, included sociodemographic data, health-related variables and theoretical variables including beliefs about breast cancer and mammography screening.

Sociodemographic data were obtained at baseline on age, education, income, insurance status, marital status, race and ethnicity. Perceived financial adequacy was measured with a single item assessing participants’ ability to pay their bills. Rurality and socioeconomic deprivation were obtained from addresses provided by the participants. Rural Urban Commuting Area (RUCA) codes were used to assess rurality [27]. Socioeconomic deprivation was measured using the 2019 Area Deprivation Index (ADI) at the national level (Range 1–100), with 100 being considered highest disadvantage [28]. Another deprivation measure specific to cancer, the Yost Index (Range 1–5), was calculated. The Yost Index provides a socioeconomic status score (range 1–5) where higher values correspond to greater affluence and is derived from seven SES-related variables from the American Community Survey [29,30]. 

Theoretical variables included questions about provider recommendation for breast cancer screening, reminder prompts for breast cancer screening sent from a health care facility, screening status for other cancers they were eligible to receive including cervical and CRC, smoking history, and height and weight. Each participant’s body mass index (BMI) was calculated from height and weight according to the Center for Disease Control and Prevention categories. 

Health beliefs included beliefs such as perceived susceptibility, self-efficacy, perceived benefits and barriers to mammography screening, and knowledge about risk of breast cancer. Stage of adoption was measured as a no/yes response to whether individuals planned to obtain a breast cancer screening in the next six months (precontemplation/contemplation) [31]. Perceived barriers were measured with nine questions anchored with Likert scales (strongly agree = 1 to strongly disagree = 5) and then summed into a mammography scale for barriers (range 9–45). Perceived benefits of breast cancer screening were assessed with a single question related to how breast cancer screening would prevent participants from worrying about dying of breast cancer. Perceived self-efficacy was assessed with one question about their confidence in obtaining a breast cancer screening. Perceived risk of breast cancer was measured with a single question pertaining to how likely a woman was to get each cancer in her lifetime compared to other women (“higher”, “about the same”, “lower”). Breast cancer and breast cancer screening knowledge (range 0–5) was assessed with 5 multiple choice and true/false questions that were coded as either correct or incorrect and then summed. These scales have been previously validated [19].

All potentially eligible women gave informed consent for inclusion before participating in the study. The study was approved by the Institutional Review Boards of Ohio State University (primary) and Indiana University (secondary) and was registered on clinical trials.gov (ID: NCT02795104). Design, recruitment and baseline characteristics have been described previously [32].

### 2.4. Statistical Approach

Sample and power calculations were based on the primary analyses of being UTD for screening for all three cancers [33]. Bivariate analyses were performed with the two-sided Fisher’s exact test for categorical variables and ANOVA for continuous variables. Baseline characteristics were descriptively reported for the overall sample and separately for women in each of the three groups [34]. 

Baseline characteristics that were marginally bivariately associated (*p* < 0.25; to provide conservative adjustment and unbiased efficacy estimation) with the outcome of UTD for breast cancer screening. were entered into the first step of a multivariable backward-deletion logistic regression procedure to compare study groups on the outcome, while adjusting for potentially confounding covariates. The Akaike Information Criterion (AIC) [35] was used as the criterion for backward removal and final model selection. Covariates of study group, age and baseline status for all eligible screening tests were forced into all models. The final model was re-run using only variables retained in the backward-removal selection to reduce missing data.

## 3. Results

**Figure 1:**
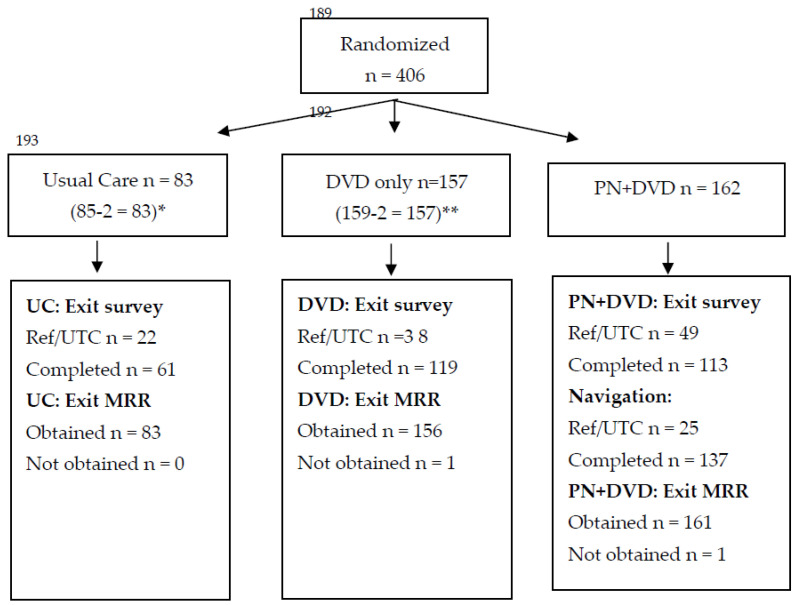
CONSORT Diagram. * Post randomization, 2 participants from Usual Care were ineligible: One person had no 12-month medical record or self-report outcome data, and one person was ineligible because it was determined that they were UTD with all screenings at baseline. ** Post randomization, 2 participants from the DVD group were determined to be ineligible because they were UTD with all screenings at baseline.

A total of 402 eligible women, from 96 of the 98 rural counties, were not UTD with mammography screening and were randomized to UC, DVD or DVD/PN intervention arms (Figure 1). Women ranged in age from 50 to 74 with a mean of 58.2 years. High school education or less was reported by 17%, 38% had some college, 27% indicated a four-year college degree and 18% reported a master’s degree or higher. A total of 97% reported being White; most women were married or living with a partner (76%), 21% were divorced or widowed, and 3% had never been married. Only 20% of women reported a household income under USD 40,000, 40% reported an income of USD 40,000 to USD 79,999 and 37% indicated a household income of USD 80,000 or greater. The majority (58%) of participants reported that they had enough money to pay their bills and money for special things while 32% reported they could pay their bills but have little extra money. Baseline data reveled little missing data except for height and weight where 38% had missing data, preventing calculation of BMI for these participants. Baseline characteristics were balanced between the three randomized arms, as expected (Table 1).

Approximately 30% of women randomized to both UC and the DVD alone groups received a mammogram by T3 (Table 2). A total of 54% of women in the combined intervention (DVD + PN) group had a mammogram within the 12-month window, with 5 times greater odds than the UC group of being UTD for breast cancer screening at T3, after adjusting for covariates (Table 3; OR = 5.11; 95% CI = 2.57, 10.60; *p* < 0.001). The DVD alone was not significantly better than UC, but the effect size was in the anticipated direction (OR = 1.26). A second analyses tested all intervention and covariate interactions at the 0.01 alpha level, and none were significant, thus the magnitude or direction of the intervention effects did not depend on any of the measured baseline covariates.

Predictors of being UTD were assessed with multivariable models. Participants were significantly less likely to be UTD at T3 if they had difficulty paying bills (OR = 0.24, *p* = 0.008) and marginally less likely to be UTD if they had a higher ADI (OR = 0.98, *p* = 0.051). Participants were significantly more likely to be UTD at 12 months if they were employed either part time (OR= 2.13, *p* = 0.038) or full time (OR = 1.89, *p* = 0.046). If women had received reminders for mammography (OR = 1.76, *p* = 0.030) or were planning at baseline to have a mammogram (OR = 1.85, *p* = 0.028), they were almost two times more likely to be UTD with breast cancer screening guidelines at T3.

Our final analyses sought to determine if there was an association between the type of screening needed (not UTD) at baseline (breast, cervical or colon) and being UTD at T3 with breast cancer screening using more data collected in the larger study. Of the 402 women who needed mammography, 59 (14.6%) were not UTD for mammography alone, 68 (16.9%) were not UTD for both breast and cervical cancer, 89 (22.1%) were not UTD for both breast and CRC, and 186 (46.3%) were not UTD for all three screenings. If women were not UTD with breast cancer screening, but UTD with cervical and CRC screening, they were 2.5 times more likely to obtain a mammogram at 12 months compared to women who were not UTD with screening for all three cancers (Table 3; OR = 2.55, *p* = 0.016).

## 4. Discussion

This study sought to determine the comparative effectiveness of two tailored interventions delivered to rural women’s homes. Both involved a tailored DVD, with one also including PN, which allowed for assessment and an individualized approach to resolving individual barriers to screening. Results demonstrated that women receiving the tailored DVD followed by telephone based PN were over five times more likely to become UTD with mammography screening compared to women randomized to UC, consistent with other studies that have tested some combination of tailored messaging and PN [19,20]. For example, a meta-analysis found that interventions led by PNs significantly increased mammography, almost twofold over UC [36]. PN has been proven effective in addressing not only lack of screening for multiple cancer sites, but also was shown to improve follow-up after positive screens [37]. The success of PN mainly comes from the approach—assess and resolve barriers to the specific action—and the delivery mechanism—usually a person who is from the same community as the participant. 

This study extended the way effective PN can be delivered—remotely over the phone—addressing unique barriers to care rural women face, e.g., transportation, low access to care, and large distances to travel. Moreover, with the digital divide, the phone as an option to provide PN was a necessity, given the difficulty with telehealth in many rural areas [38]. Several covariates significantly impacted mammography screening (Table 3). First, financial status affected becoming UTD with breast cancer screening. Compared to those who had enough money for special things after paying their bills, those who had difficulty paying bills [28,39] were much less likely to get a mammogram (OR = 0.24, *p* = 0.008) as found in other studies [39,40]. Consistent with this finding, those who evidenced greater area deprivation, as measured by the ADI [39,40] were less likely to become UTD with mammography. Perhaps women were not aware that Medicaid, Medicare and all insurance companies are required to provide mammograms with no out of pocket payments or that many states, including OH and IN, participate in the Breast and Cervical Cancer Early Detection Program, which allows women who qualify to obtain free or low cost mammograms [41,42]. Given the wide variety of resources that ensure no out of pocket expenses for mammography, the availability of financial support for mammography needs to be emphasized to women with financial concerns.

Both receiving a reminder about mammography and voicing intent to screen in the next six months significantly predicted obtaining mammography, a finding that has been reported in other studies [8,43]. Women who only needed breast cancer screening at baseline compared to those who were not UTD with two or more screenings were more likely to obtain a mammogram by T3. While it is obviously easier to obtain a single needed screening compared to multiple tests, our interventions [33] sought to motivate women to become UTD with any needed screenings. Mammography was the most accepted of the three screenings by the participants, perhaps because of widespread acceptability and promotion of breast cancer screening compared to the other two tests and the nature of the tests themselves, with mammography being less invasive than Pap tests and colonoscopies and perhaps less off-putting than completing a Fecal Immunochemical Test (FIT).

## 5. Strengths

We obtained eligibility status (at baseline), and T3 outcome data through MRR. We confirmed medical record home again at T3. We were unable to reach about 10% of our sample to confirm the location of their current medical records and used their medical record provided at baseline. If women had changed health care locations, a mammogram obtained after the intervention might not be recorded in the baseline medical record system, thus underestimating mammography receipt. However, we were able to control for this difference with a variable indicating if MRR was confirmed at T3. We used well-validated survey measures to capture our variables of interest. Our interventions were based on prior work, and we assessed the robustness of intervention delivery. Importantly, o interventions were tailored to the needs of this rural population.

## 6. Limitations

Although participants were recruited from rural counties in Ohio and Indiana, the overall socioeconomic status of our sample was high, thus, our sample might not be truly representative of women in these counties [5,44]. Additionally, although eligibility was defined as living in counties with RUCC 4–9, women may have been more aligned with neighboring urban counties and not representative of rural counties with smaller populations. Lastly, we might have underestimated screening for some women, as medical records might not have captured all tests received at other clinical locations.

## 7. Conclusions

For women who were not UTD with mammography screening at baseline, women in the DVD/PN intervention group compared to women in the UC group were over five times more likely to become UTD with breast cancer screening. Although there were not significant interactions with the intervention, we found that being in contemplation (intending to have a mammogram in the next 6 months), being UTD with cervical and colon cancer screenings, having more disposable income and receiving a mammography reminder were associated with becoming UTD with breast cancer screening. We also found that women who lived in areas with greater socioeconomic deprivation were less likely to become UTD with breast cancer screening, supporting the need for adapting interventions so they can be delivered remotely to women in deprived areas. The ability of this intervention to be delivered remotely to women in rural areas will allow mammography-supporting interventions to be disseminated to women in the most underserved and under-resourced areas of the United States.

## Figures and Tables

**Table 1:** Baseline characteristics by randomized study group.

Characteristic	Overall N = 402 ^1^	Usual Care N = 83 ^1^	DVD N = 157 ^1^	DVD/PN N = 162 ^1^
**Outside Guidelines For:**				
All 3 tests	186 (46%)	37 (45%)	75 (48%)	74 (46%)
Mammogram & CRC	89 (22%)	18 (22%)	34 (22%)	37 (23%)
Mammogram & cervical cancer	68 (17%)	15 (18%)	26 (17%)	27 (17%)
Mammogram only	59 (15%)	13 (16%)	22 (14%)	24 (15%)
**Age**	58.2 (6.1)	58.7 (6.0)	58.0 (6.2)	58.1 (6.0)
50–54	140 (35%)	25 (30%)	55 (35%)	60 (37%)
55–59	109 (27%)	21 (25%)	49 (31%)	39 (24%)
60–64	82 (20%)	21 (25%)	27 (17%)	34 (21%)
65+	71 (18%)	16 (19%)	26 (17%)	29 (18%)
**State**				
Indiana	168 (42%)	36 (43%)	67 (43%)	65 (40%)
Ohio	234 (58%)	47 (57%)	90 (57%)	97 (60%)
**Education**				
HS/GED or less	69 (17%)	17 (20%)	26 (17%)	26 (16%)
Some college or AS	154 (38%)	29 (35%)	64 (41%)	61 (38%)
BS/BA/AB/BSN	108 (27%)	24 (29%)	41 (26%)	43 (27%)
MS or more	71 (18%)	13 (16%)	26 (17%)	32 (20%)
**Income**				
<USD 40 k	80 (20%)	22 (27%)	33 (21%)	25 (15%)
USD 40k–USD 79,999	159 (40%)	27 (33%)	65 (41%)	67 (41%)
USD 80 k +	150 (37%)	28 (34%)	58 (37%)	64 (40%)
Missing	13 (3.2%)	6 (7.2%)	1 (0.6%)	6 (3.7%)
**Marital Status**				
Married/living as married	303 (76%)	62 (76%)	120 (76%)	121 (75%)
Divorced/Widowed/Separated	86 (21%)	19 (23%)	33 (21%)	34 (21%)
Never married	12 (3.0%)	1 (1.2%)	4 (2.5%)	7 (4.3%)
**Insurance Status**				
Private only	267 (67%)	57 (69%)	101 (65%)	109 (67%)
No insurance	37 (9.2%)	10 (12%)	17 (11%)	10 (6.2%)
Public only	43 (11%)	9 (11%)	16 (10%)	18 (11%)
Public and private	54 (13%)	7 (8.4%)	22 (14%)	25 (15%)
White	391 (97%)	80 (96%)	156 (99%)	155 (96%)
Non-White	11 (2.7%)	3 (3.6%)	1 (0.6%)	7 (4.3%)
**Household Financial Situation**				
Has enough money for special things	231 (58%)	46 (55%)	96 (61%)	89 (55%)
Can pay bills, but little extra money	127 (32%)	28 (34%)	46 (29%)	53 (33%)
Has to cut back or has difficulty paying bills	43 (11%)	9 (11%)	15 (9.6%)	19 (12%)
National Percentile of Block Group ADI Score	67.8 (15.9)	69.8 (16.0)	66.6 (16.3)	67.9 (15.4)
**Secondary RUCA Code**				
Urban and Large Rural City/Town	257 (64%)	54 (65%)	104 (66%)	99 (61%)
Small and Isolated Small Rural Town	145 (36%)	29 (35%)	53 (34%)	63 (39%)
**Yost—U.S.-based, Quintiles**				
1—Lowest SES	60 (17%)	14 (19%)	16 (11%)	30 (20%)
2	161 (44%)	30 (40%)	66 (47%)	65 (44%)
3	112 (31%)	22 (29%)	46 (33%)	44 (30%)
4 or 5—Highest SES	30 (8.3%)	9 (12%)	12 (8.6%)	9 (6.1%)
**Working for Pay**				
No	135 (34%)	30 (36%)	54 (34%)	51 (31%)
Yes—part time	83 (21%)	22 (27%)	27 (17%)	34 (21%)
Yes—full time	184 (46%)	31 (37%)	76 (48%)	77 (48%)
**Smoking Status**				
Never	247 (61%)	56 (67%)	95 (61%)	96 (59%)
Former	112 (28%)	17 (20%)	47 (30%)	48 (30%)
Current	31 (7.7%)	7 (8.4%)	11 (7.0%)	13 (8.0%)
**Body Mass Index (BMI)**				
Obese	129 (32%)	26 (31%)	49 (31%)	54 (33%)
Normal	50 (12%)	15 (18%)	20 (13%)	15 (9.3%)
Overweight	72 (18%)	16 (19%)	25 (16%)	31 (19%)
Unknown	151 (38%)	26 (31%)	63 (40%)	62 (38%)
**Ever Had a Mammogram**				
No	30 (7.5%)	4 (4.8%)	12 (7.6%)	14 (8.6%)
Yes	372 (93%)	79 (95%)	145 (92%)	148 (91%)
**Health Care Provider Suggested to Have a Mammogram**				
No	21 (5.3%)	2 (2.4%)	9 (5.8%)	10 (6.2%)
Yes	375 (95%)	80 (98%)	145 (94%)	150 (94%)
**Received Reminders from Health Care Facility**				
No	208 (54%)	40 (49%)	87 (59%)	81 (52%)
Yes	176 (46%)	41 (51%)	61 (41%)	74 (48%)
**Planning to Have a Mammogram in Next 6 Months**				
No	207 (51%)	40 (48%)	78 (50%)	89 (55%)
Yes	195 (49%)	43 (52%)	79 (50%)	73 (45%)
**Perceived Barriers to Mammography Screening Score** (range: 9–45)	20.2 (5.2)	19.6 (4.8)	19.8 (5.4)	20.8 (5.3)
**If Have Regular Mammograms, Won′t Worry as Much about Dying from Breast Cancer**				
Neither/Disagree/strongly disagree	146 (37%)	23 (28%)	54 (35%)	69 (43%)
Strongly agree/agree	253 (63%)	60 (72%)	101 (65%)	92 (57%)
**Compared to Women Your Age and Race, How Likely to Get Breast Cancer**				
About the same	249 (62%)	53 (64%)	90 (58%)	106 (66%)
Higher	32 (8.0%)	8 (9.6%)	10 (6.5%)	14 (8.7%)
Lower	118 (30%)	22 (27%)	55 (35%)	41 (25%)
**Confident Can Get a Mammogram**				
Neither/Disagree/strongly disagree	46 (12%)	11 (13%)	14 (9.0%)	21 (13%)
Strongly agree/agree	353 (88%)	72 (87%)	141 (91%)	140 (87%)
**Breast Cancer Knowledge Score** (range: 0–5)	3.38 (1.14)	3.22 (1.23)	3.54 (1.09)	3.31 (1.13)

^1^ n (%); Mean (SD).

**Table 2:** Bivariate analysis of 12-month medical record breast cancer screening outcome by study arm (N = 402).

	Randomized Arm				*p*-Values			
Characteristic	Overall, N = 402 ^1^	Usual Care ^1^	DVD ^1^	DVD/ Navigator ^1^	*p*-Value ^2^	DVD vs. Usual Care ^3^	DVD/Navigator vs. Usual Care ^3^	DVD/Navigator vs. DVD ^3^
**UTD for breast cancer screenings within 12 months since enrollment**					<0.001	>0.9	<0.001	<0.001
**No record of test or outside 12-month window**	243 (60%)	58 (70%)	110 (70%)	75 (46%)				
**Received within 12 months**	159 (40%)	25 (30%)	47 (30%)	87 (54%)				

^1^ n (%) ^2^ Pearson’s Chi-squared test ^3^ Fisher’s exact test. Note: UTD = up to date.

**Table 3:** Multivariable logistic regression model of being UTD for breast cancer screening at 12 months (N = 355).

Characteristic	OR ^1^	95% CI ^1^	*p*-Value
**Baseline screening status, not UTD for:**			
Breast, colorectal and cervical	—	—	
Breast and colorectal	1.55	0.79, 3.04	0.202
Breast and cervical	1.84	0.91, 3.75	0.091
Breast only	2.55	1.20, 5.49	**0.016**
**Study arm ^2^**			
Usual care	—	—	
DVD	1.26	0.64, 2.52	0.503
DVD/Patient Navigator	5.11	2.57, 10.60	**<0.001**
**Age**			0.412
50–54	—	—	
55–59	1.31	0.67, 2.55	0.433
60–64	1.86	0.90, 3.89	0.094
65+	1.43	0.64, 3.20	0.381
**Describe your household financial situation**			
Has enough money for special things	—	—	
Can pay bills, but little extra money	0.87	0.49, 1.52	0.621
Has to cut back or has difficulty paying bills	0.24	0.08, 0.65	**0.008**
**Currently working for pay**			
No	—	—	
Yes—part time	2.13	1.04, 4.39	**0.038**
Yes—full time	1.89	1.02, 3.58	**0.046**
**Received any mammogram reminders**			
No	—	—	
Yes	1.76	1.06, 2.94	**0.030**
**Planning to have a mammogram in the next 6 months**			
No	—	—	
Yes	1.85	1.07, 3.22	**0.028**
**If have regular mammograms, won′t worry as much about dying from breast cancer**			
Neither/Disagree/strongly disagree	—	—	
Strongly agree/agree	1.76	0.99, 3.17	0.057
**National percentile of block group ADI score**	0.98	0.97, 1.00	0.051

^1^ OR = Odds Ratio, CI = Confidence Interval. ADI = area disadvantage index. UTD = up to date. ^2^ Effect of DVD/Navigator vs. DVD: OR = 4.05; 95% CI = 2.26, 7.26; *p* < 0.001. Notes: The omnibus tests are likelihood ratio tests and may therefore disagree slightly with the Wald tests for specific indicator variables. *p*-values < 0.05 are bolded. Re-confirmation of medical record location at 12 months was also adjusted for in this model (confirmed vs. not confirmed at 12 months; OR = 4.60; CI = 2.14, 10.70, *p* < 0.001); for the 12% of participants whose medical record health care system location was not confirmed at 12 months, the location reported at their baseline interview was used to assess 12-month outcomes; in the total sample, only 5 persons had no medical record data or location confirmation at baseline or 12 months, and, among those 5, the 12-month self-report data was available and used for 4 persons in all analyses.

## Data Availability

The data can be shared upon request addressed to vchampio@iu.edu.
